# Promoting Pedagogical Resilience: Unveiling the Efficacy of Synchronous Online Lectures Compared to Traditional Methods From the Students’ Vantage Point Amidst the COVID-19 Pandemic

**DOI:** 10.7759/cureus.68391

**Published:** 2024-09-01

**Authors:** Smita R Sorte, Sachin B Rathod, Dipali K Chatur, Anup Kumar D Dhanvijay

**Affiliations:** 1 Physiology, All India Institute of Medical Sciences, Nagpur, Nagpur, IND; 2 Physiology, All India Institute of Medical Sciences, Raipur, Raipur, IND; 3 Physiology, All India Institute of Medical Sciences, Deoghar, Deoghar, IND

**Keywords:** synchronous online learning, synchronous learning, online medical education, online teaching, covid 19 pandemic, covid 19, classroom learning, classroom teaching, online synchronous

## Abstract

Introduction

The COVID-19 pandemic significantly disrupted traditional educational methods, forcing medical institutes to adapt to online classes. Since online teaching was an untested approach in Indian medical education, student feedback was essential. This study compares synchronous online lectures with traditional classroom lectures from the students' perspective.

Method

The cross-sectional study was conducted on undergraduate medical students across India undergoing synchronous online MBBS classes during the COVID-19 pandemic. After obtaining institutional ethical clearance, five-section questionnaires via Google Forms were circulated on WhatsApp and Facebook. The generated quantitative data were analyzed using descriptive statistics and frequency analysis.

Result

Out of 341 responses, 64 students did not consent and were excluded, resulting in 277 responses for analysis. The total score for traditional face-to-face lectures was 11.61 out of 15, with a percentage mean score of 77%, highlighting the significant positive influence of instructor presence and a conducive learning environment. In contrast, synchronous online lectures had a total score of 16.01 out of 25, with a percentage mean score of 64.04%, reflecting mixed responses. The Student's t-test analysis underscored the significant advantages of face-to-face interactions and structured learning environments in traditional classroom settings despite the flexibility and independence offered by synchronous online lectures.

Conclusion

The findings of this study reveal that students have a moderate level of satisfaction with synchronous online lectures, as indicated by a mean score of 64.04%. While these lectures provide flexibility and promote independence, they require students to exhibit higher levels of self-motivation, discipline, and self-directed learning. However, the online format poses challenges for effective communication and technical issues. Addressing the challenges of technology use, teacher training, and student engagement can enhance the effectiveness of online teaching and ensure that it complements traditional teaching methods, ultimately promoting pedagogical resilience in medical education.

## Introduction

In the late 19th and early 20th centuries, before the advent of the digital age, distance learning primarily took the form of correspondence courses. This evolved to include educational broadcasts via radio and television, serving as early precursors to modern e-learning.

By the mid to late 1990s, distance or asynchronous teaching began to gain traction in the United States [1], a trend that was significantly accelerated with the introduction of Massive Open Online Courses (MOOCs) in 2012 [2]. These developments were marked by a growing online presence and expanded opportunities for open learning.

Despite these advancements, higher education institutions still view e-learning as a complement rather than a replacement for traditional instructor-led training.

Before the COVID-19 pandemic in India, medical education predominantly relied on physical, face-to-face interactions, including traditional lectures, small group discussions, tutorials, practical sessions, and clinical exposure in hospitals, as recommended by the National Medical Council for undergraduate medical training [3].

The onset of the COVID-19 pandemic in early 2020 led to widespread lockdowns, including in India, where a nationwide lockdown was implemented on March 25, 2020. This abrupt disruption forced a sudden shift from traditional in-person teaching to online learning, with medical schools rapidly adopting digital platforms to continue education. This transition marked one of the most significant changes in the history of medical education, as institutions worldwide overcame established barriers to implement online curricula [4-7].

In response to this shift, the National Medical Council of India issued guidelines and recommendations for conducting online classes [3]. However, only a few institutions had the facilities and trained faculty to deliver online education effectively. As online teaching was a relatively new approach in Indian medical education, gathering student feedback to assess its effectiveness became imperative.

This study aims to compare synchronous online lectures with traditional classroom-based lectures from the students' perspective. The research question for this study is: "How do students perceive the effectiveness and impact of synchronous online lectures compared to traditional classroom-based teaching in the context of medical education?"

Understanding how students perceive this shift is crucial for several reasons. First, it provides insights into the strengths and weaknesses of synchronous online teaching compared to traditional methods, which have long been the cornerstone of medical education. Second, this understanding is essential for refining online teaching methods, making them more effective and engaging for students. Lastly, the findings of this study could inform higher education bodies, such as the National Medical Council of India, in revising and enhancing guidelines for online medical education, ensuring that it complements traditional learning and meets the needs of the future healthcare workforce.

## Materials and methods

We obtained approval from the All India Institute of Medical Sciences (AIIMS), Nagpur, and the Institutional Ethics Committee (approval number: IEC/Pharmac/86/2020). The study was conducted over three months (May-July 2020).

The cross-sectional study was conducted on undergraduate medical students across India undergoing synchronous online MBBS classes during the COVID-19 pandemic. The consent process was conducted online using Google Forms, where students were informed about the study's purpose. Participants were explicitly informed that their participation was voluntary and that they could withdraw from the study at any time without any consequences.

Two medical educators, based on their expertise in medical education and online learning, designed the questionnaires. The questionnaire underwent pilot testing with 20 medical students and achieved a Cronbach's alpha score of 0.840 for reliability. Medical educators conducted expert reviews to ensure content validity. The outcomes of the pilot testing were carefully analyzed for clarity and relevance based on student feedback.

Questionnaires

The survey had five parts as follows:

Section A: Participant Information and Consent

The study aims were explained, and students were asked to provide consent. Students were asked to provide their email, age, year of MBBS (1st, 2nd, 3rd, or 4th year), and gender.

Section B: Internet Access and Digital Literacy

The questionnaire included questions about internet access and device ownership to assess the students' access to technology and their proficiency with digital tools. Students indicated their method of internet access (cellular internet, broadband, Wi-Fi, or dial-up connection) and their devices (laptop, desktop, smartphone, or iPhone). Additionally, the questionnaire evaluated their digital literacy by asking if they were familiar with learning management systems and basic computing programs, and if they felt comfortable helping others troubleshoot technical issues. They rated their digital literacy and tech-savviness on a scale of 'No,' 'Maybe,' or 'Yes.'

Section C: Questions About Traditional Classes

Students rated the following statements on a five-point Likert scale from 1 (Strongly Disagree) to 5 (Strongly Agree) to assess their perceptions of traditional classroom settings:

The impact of the instructor's physical presence on their learning and motivation, the influence of the classroom environment and presence of peers on their active learning, and their sense of discipline and activeness when attending classroom lectures.

Section D: Questions About Synchronous Online Classes

Students rated their experiences with synchronous online classes on a similar five-point Likert scale from 1 (Strongly Disagree) to 5 (Strongly Agree).

Whether the instructor's lack of physical presence benefited their learning, attending lectures alone in a room provided more freedom and stimulated learning, the need for increased motivation, discipline, and self-direction in synchronous online lectures, online courses felt more personalized and individualized compared to face-to-face settings, difficulty in asking questions to online instructors due to impersonal communication.

Section E: Comparison of Traditional and Synchronous Online Methods

Students were asked to compare various aspects of traditional face-to-face lectures and synchronous online video lectures. They indicated their preferences for each method or both for the following criteria: concentration span, providing enough time for taking notes, the feasibility of asking questions in real-time, class engagement, resource utilization, providing immediate feedback, ease of giving class attendance, making them feel more responsible regarding exams, studying at their own pace, distractions, enthusiasm and interest, memory retention, subject understanding, preference for theory lectures, practical classes, tutorials, and case discussions.

This comprehensive questionnaire aimed to capture a detailed understanding of medical students' preferences and experiences with traditional and synchronous online learning methods.

Survey dissemination

The questionnaires were circulated via Google Forms on Facebook, WhatsApp, and email to all college students. The forms were also sent to faculties of various colleges to be circulated among their students. The survey was disseminated over one month to ensure adequate participation from medical students across various years of study (1st, 2nd, 3rd, and 4th year). Reminders were sent after 7 days to encourage participation and maximize response rates. Convenience sampling was used.

Data segregation and analysis

We included only students who had previously attended traditional face-to-face classes and were presently attending online and synchronous courses. Students taking asynchronous classes or using any other e-learning method were excluded from the study. Questions with response options "No," "Not sure," and "Yes" were scored 1, 2, and 3, respectively. Questions with five response options were scored from 1 to 5, where 1 represented the lowest grade (Strongly Disagree) and 5 represented the highest grade (Strongly Agree). The mean score for each question was calculated to determine the students' average response. For each domain, the mean scores of all related questions were summed to obtain a total score, which was then converted into a percentage for easy interpretation and comparison across domains.

Data analysis

Data were entered using Microsoft Excel (Microsoft Corp., Redmond, WA), and analysis was conducted with Jamovi open statistical software version 2.3.28 (released in 2024). We applied descriptive statistics to present results in terms of numbers and percentages. The average score for each 3-point and 5-point question was computed to gauge the students' responses. These scores were then converted into percentages to simplify. This normalization process facilitated a more straightforward and intuitive interpretation of the results. Group differences in scores were evaluated using the Student's t-test, with p-values below 0.05 considered statistically significant.

## Results

Out of 341 responses received, 64 students did not give consent and were excluded, resulting in 277 responses for analysis. Among these, 151 (54.5%) were male and 126 (45.5%) were female. The maximum response was obtained from the third year (126, 45.5%), followed by the first year MBBS (81, 29.2%), the second year MBBS (66, 23.8%), and lastly, from the final year (4, 1.4%).

Of the total students, 219 (79.1%) accessed the internet via cellular networks, 27 (9.7%) used Wi-Fi, 4 (1.4%) had broadband connections, and 6 (2.2%) relied on dial-up connections. Out of the students surveyed, 211 (76.47%) use smartphones, 46 (16.7%) use multiple devices, 11 (4%) use laptops, 7 (2.5%) use iPhones, and 2 (0.4%) use desktops.

**Figure 1:**
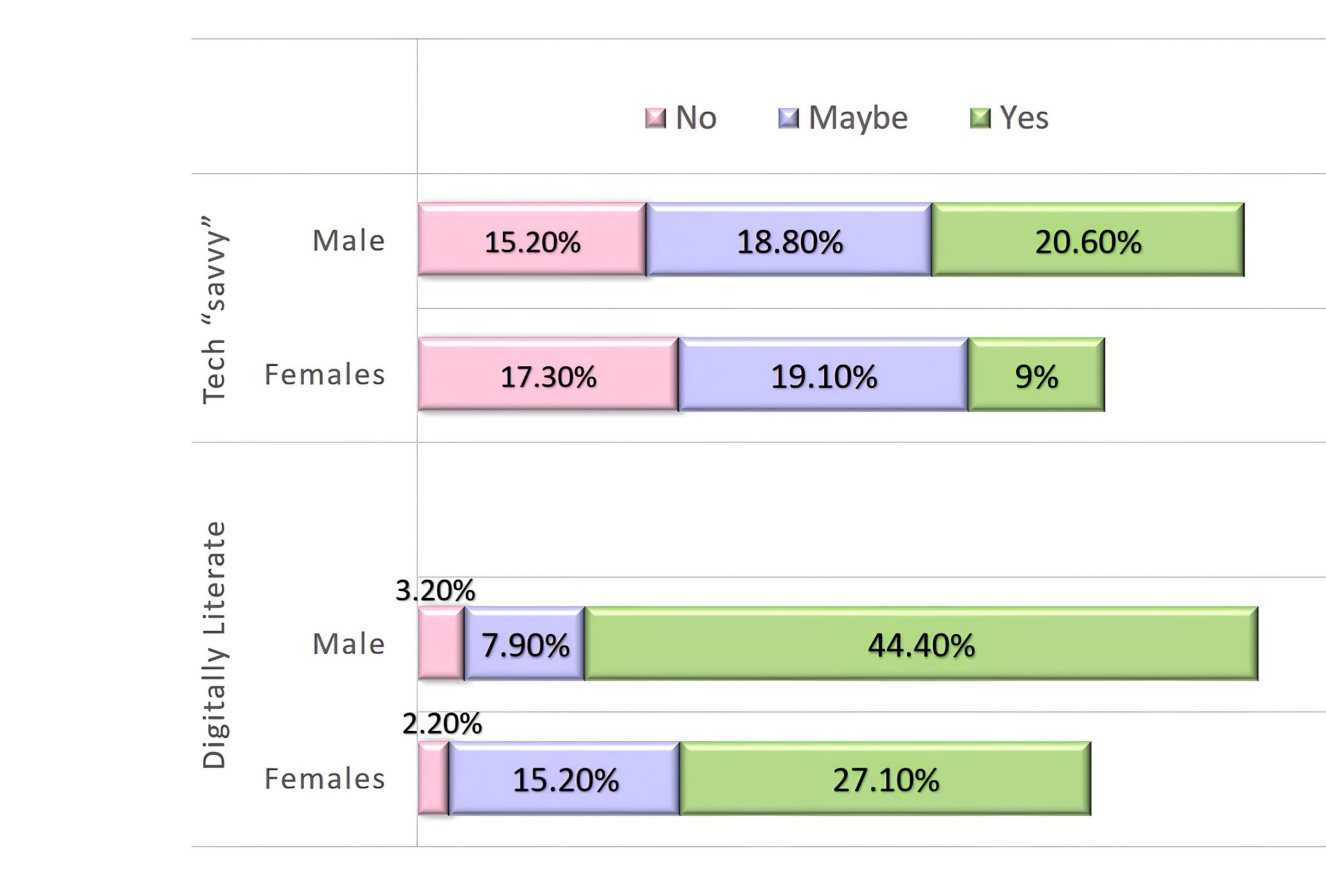
Percentage of students who are tech-savvy and digitally literate.

Regarding digital literacy, the results indicate that most students have a high level of proficiency. A total of 198 students (71.5%) were digitally literate, meaning they knew how to use learning management systems and basic computing programs such as email, Google apps, and publisher software (such as Word). They were comfortable helping other students troubleshoot fundamental technical difficulties. Meanwhile, 64 (23.1%) were unsure, and only 15 (5.4%) of the students were digitally illiterate. Regarding tech savviness, 90 (32.5%) students were not experts in using computers or smartphone information technology, 105 (37.9%) were not sure, and 82 (29.6%) considered themselves tech-savvy (Figure 1).

For digital literacy questions, response options were 'No,' 'Not sure,' and 'Yes,' which were scored 1, 2, and 3, respectively. A mean score of 2.66 and a standard deviation of 0.578 was obtained, corresponding to a percentage score of 88.67%, indicating the students' high level of digital literacy.

In contrast, the parameter assessing tech savviness, with scores ranging from 1 to 3, had a mean score of 1.97, with a standard deviation of 0.789, resulting in a percentage score of 65.67%. This percentage reflects a comparatively moderate level of technological proficiency among the students (Table 1).

**Table 1:** Descriptive statistics for students' digital skills parameters.

Parameters	N	Minimum	Maximum	Mean	SD	Percentage
Digital literacy	277	1	3	2.66	0.578	88.67%
Tech “savvy”	277	1	3	1.97	0.789	65.67%

The survey on traditional face-to-face lectures reveals student perceptions of their learning environment. Out of the total respondents, the instructor's presence significantly affects learning and motivation, with a mean score of 3.79 and a standard deviation of 1.09 from 274 responses. Similarly, the learning environment, including the classroom setting and the presence of fellow students, positively impacts active learning, as indicated by a mean score of 3.78 and a SD of 1.28 from 272 responses.

Furthermore, students feel more disciplined and physically active when attending classroom lectures, with this aspect receiving the highest mean score of 4.04 and a standard deviation of 1.14, also from 272 responses. The total score for these aspects of traditional face-to-face lectures is 11.61 out of 15, resulting in a percentage mean score of 77%.

These results underscore the significant positive influence of instructor presence, a conducive learning environment, and the traditional classroom setting on students' learning experiences and discipline (Table 2).

**Table 2:** The student’s perception of traditional face-to-face lectures.

Sr. No.	Questions on Traditional Face-to-Face Lectures	N	Missing	Mean	SD	Minimum	Maximum	One Sample T-test P-value
1	The instructor's presence affects your learning and motivates you to learn	274	3	3.79	1.09	1	5	<0.01
2	The learning environment (classroom and presence of colleagues) affects your active learning	272	5	3.78	1.28	1	5	<0.01
3	Do you feel more disciplined and physically active in attending classroom lectures?	272	5	4.04	1.14	1	5	<0.01
	Total Score			11.61	3.51		15	
	Percentage Mean Score			77%			100	

The survey results regarding online synchronous lectures offer a nuanced view of student experiences. The instructor in an online setting is perceived as moderately beneficial, with a mean score of 2.88 and a standard deviation of 1.26 (P<0.001) from 268 responses. Students feel that attending lectures alone in a room gives them more freedom and stimulates their learning, as reflected by a mean score of 3.11 and a standard deviation of 1.38 (p-value < 0.001) from 274 responses.

Moreover, students indicate a need for increased self-motivation, discipline, and self-direction in online lectures, scoring this aspect a mean of 3.72 with a standard deviation of 1.23 (p-value <0.001) from 274 responses.

Regarding personal and individualized attention, students slightly favor online courses, with a mean score of 3.09 and a standard deviation of 1.36 (P<0.001) from 272 responses. However, students find it challenging to ask questions of online instructors, often perceiving communication as impersonal, as evidenced by a mean score of 3.21 and a standard deviation of 1.34 (P<0.001) from 272 responses.

Overall, the total score for these five questions is 16.01 out of a possible 25, resulting in a percentage mean score of 64.04%. These findings highlight the mixed responses towards online synchronous lectures, with students recognizing both the benefits and challenges of this learning mode (Table 3).

**Table 3:** The student’s perception of synchronous online lectures.

Sr. no	Questions on synchronous online lectures	N	Missing	Mean	SD	Minimum	Maximum	One sample T-test P value
1	The instructor not being physically present in a classroom benefits to learners attending synchronous online sessions.	268	9	2.88	1.26	1	5	<0.01
2	Attending lectures alone in a room gives me more freedom and stimulates my learning.	274	3	3.11	1.38	1	5	<0.01
3	Do you feel you need to be more motivated, disciplined, and self-directed in attending synchronous online lectures?	274	3	3.72	1.23	1	5	<0.01
4	In the synchronous online course do you feel more personal and individualized than in a face-to-face setting?	272	5	3.09	1.36	1	5	<0.01
5	Do you find it difficult to ask queries to the synchronous online instructor, as communication is often very impersonal?	272	5	3.21	1.34	1	5	<0.01
	Total Score for five questions			16.01			25	
	Percentage mean score for five questions			64.04%			100	

The analysis comparing traditional face-to-face and online synchronous lectures using the Student's T-test reveals significant differences in student perceptions and experiences across several dimensions.

Firstly, the instructor's presence in a traditional classroom setting significantly affects student learning and motivation compared to the perceived benefit of instructors in online sessions. This comparison yielded a p-value < 0.01, indicating a highly significant difference favoring traditional presence.

Secondly, the learning environment, including the classroom and the presence of colleagues, significantly enhances active learning compared to the freedom and stimulation provided by attending lectures alone in a room. This aspect produced a p-value < 0.01, again showing a significant preference for the traditional classroom environment.

Lastly, students reported feeling more disciplined and physically active when attending classroom lectures compared to the increased need for self-motivation, discipline, and self-direction in online lectures. This comparison resulted in a p-value of less than 0.01, highlighting a notable difference favoring physical attendance for promoting discipline and physical activity.

These findings underscore the significant advantages of face-to-face interactions and structured learning environments in traditional classroom settings despite the flexibility and independence offered by online synchronous lectures (Table 4).

**Table 4:** The student’s perception of traditional face-to-face lectures compared to synchronous online lectures. df: Degrees of freedom.

Sr. no	Questions on traditional face-to-face lectures	Questions on online synchronous lectures	Student t-test		
			Statistics	df	P-value
1	The Instructor's presence affects your learning and motivates you to learn.	The instructor not being physically present in a classroom benefits learners attending synchronous online sessions.	9.79	266	<0.01
2	The Learning Environment (classroom and presence of colleagues) affects your active learning.	Attending lectures alone in a room gives me more freedom and stimulates my learning.	5.76	271	<0.01
3	Do you feel more disciplined and physically active in attending classroom lectures?	Do you feel you need to be more motivated, disciplined and self-directed when attending synchronous online lectures?	3.33	271	<0.01

The survey results reveal a strong preference among students for traditional classes in various aspects of their learning experience. Specifically, 116 respondents (87.22%) prefer traditional methods for practical classes. Traditional classes are also favored for creating a comfortable learning environment (74.63%) and for case discussions (95, 71.43%). Students believe that traditional classes significantly enhance engagement (93, 69.92%) and are more effective for theory lectures (80, 60.15%).

Regarding the feasibility of asking questions in real-time, 81 students (60.66%) find traditional classes more effective. Additionally, attributes such as concentration span (161, 58.76%), enthusiasm/interest in learning (156, 57.35%), and a better understanding of the subject (153, 56.67%) are perceived to be greater in traditional settings. Furthermore, 153 respondents (56.67%) indicate that traditional classes help them memorize relevant information better.

However, when considering the feasibility of class attendance, the preference slightly tilts, with 158 respondents (54.21%) favoring traditional classes, while 33.83% find online classes more feasible for giving immediate responses. Notably, online classes are preferred for studying at one’s own pace (61.17%) and are perceived as having fewer distractions (23.08%).

**Figure 2:**
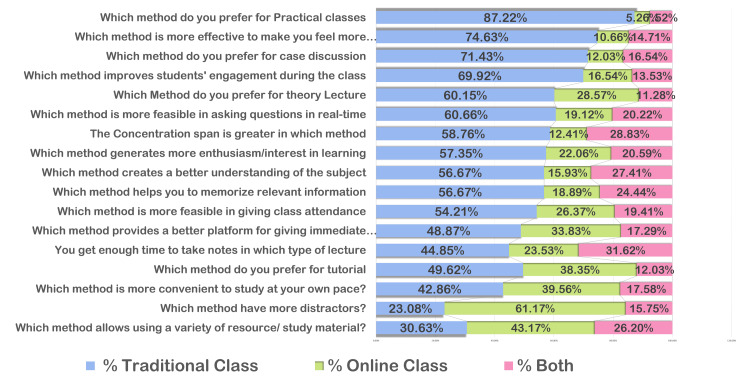
The preferred teaching method perceived by the students.

For tutorials, 66 respondents (49.62%) prefer traditional methods. Regarding the utilization of a variety of study materials, 117 students (43.17%) prefer a combination of both traditional and online methods, highlighting the importance of diverse resources in the learning process. These results indicate a predominant inclination towards traditional classes for various educational activities, with specific advantages recognized for online learning in certain aspects (Figure 2).

## Discussion

**Figure 3:**
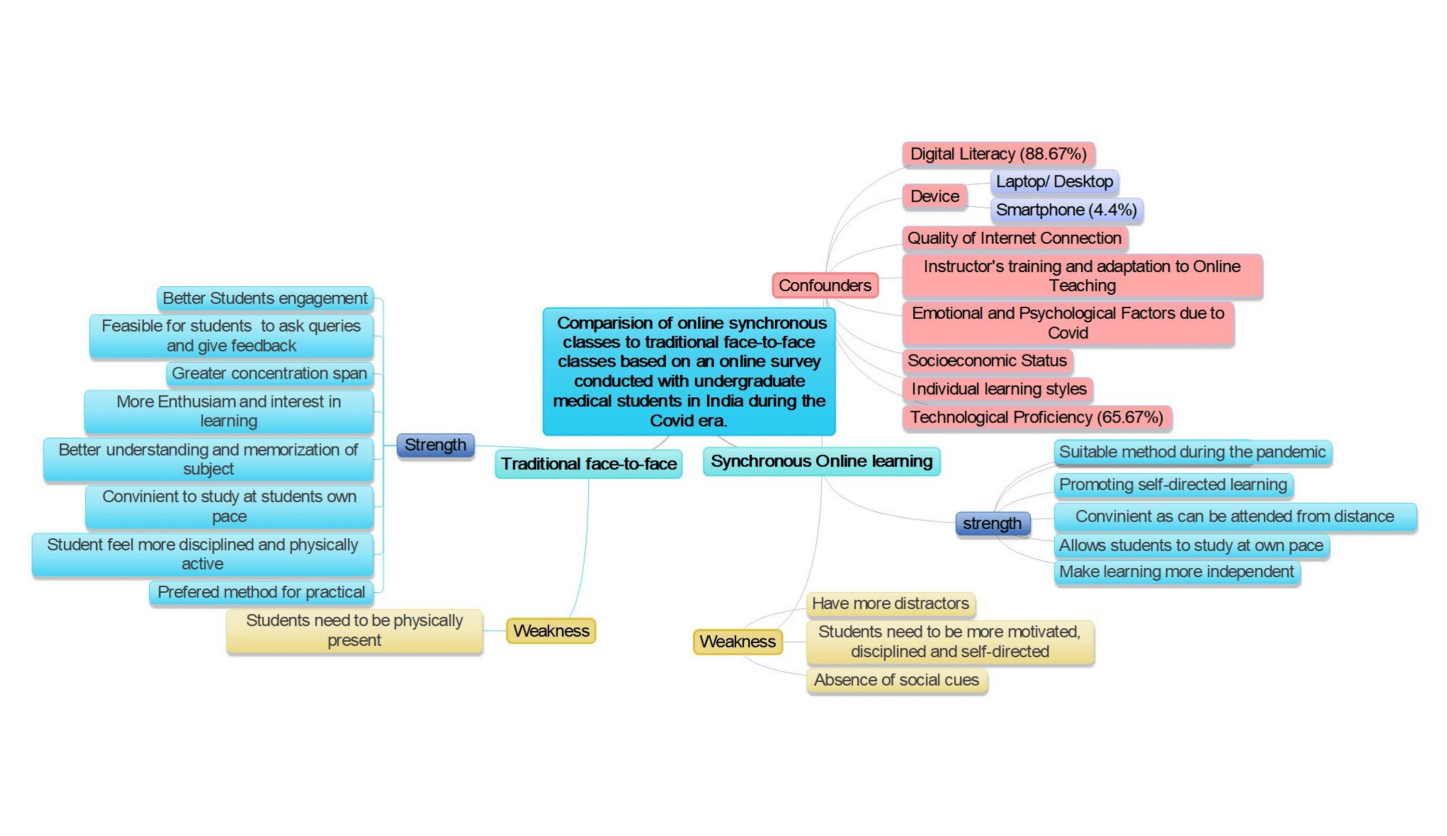
Comparison of strengths and weaknesses of synchronous online learning and traditional face-to-face classes.

The COVID-19 pandemic necessitated a rapid shift from traditional classroom lectures to synchronous online lectures, particularly in medical education. We intend to evaluate the effectiveness of online synchronous learning compared to traditional face-to-face classes, based on an online survey conducted with undergraduate medical students in India. The results provide major contributions to a better understanding of student choice, engagement, and learning outcomes in traditional versus online modes (Figure 3).

Digital literacy and technological proficiency

The survey revealed a high level of digital literacy (88.67%) and a moderate level (65.67%) of technological proficiency, suggesting that while students can navigate digital platforms, there is room for improvement, which could further enhance their online learning experience.

Only 13 (4.4%) of students could attend classes on a desktop or laptop during the COVID era, whereas the majority attended classes on their smartphones and iPhones with small screens. This could have affected the online learning experience due to digital eye strain [8] and musculoskeletal problems from faulty posture, such as a forward neck, rounded shoulders, or slouched posture [9].

Synchronous online classes: strengths and weaknesses

Synchronous online learning emerged as a pivotal educational approach during the COVID-19 pandemic, offering several distinct advantages. One of the primary strengths of this method is the increased flexibility and freedom it provides students. Unlike traditional learning environments, synchronous online classes allow students to engage in education from the convenience of their own homes, making it possible to attend classes despite geographical or situational barriers. This modality also promotes self-directed learning, as students are often required to manage their time and resources more independently.

Chalise GD et al. reported that 83.6% of MBBS students and 82.9% of B.Sc Nursing students had a positive attitude towards online classes during the COVID-19 pandemic [10].

However, this flexibility comes with significant challenges. The online environment inherently has more potential distractions compared to a traditional classroom as it relies heavily on technology, which can significantly disrupt the learning experience.

A study conducted by Sharma N et al. in Nepal found that 79.6% of students reported disturbances during online classes due to internet connectivity issues, 22.3% experienced electricity problems, and 73.5% faced external disturbances impacting their learning [11].

Shetty S et al. reported that while students favored online learning to maintain their academic interest and development during the pandemic, they faced challenges such as a lack of face-to-face interactions, socialization, distractions from social media, and technology-related issues. They preferred a combined approach to learning in the post-pandemic period [12].

Islam MI et al.'s research on dental students in Bangladesh found that 87.5% of students were dissatisfied with their online programs during the COVID-19 pandemic. The students reported not receiving assistance in overcoming barriers to accessing classes or materials (64.23%), lacked access to the institutional online learning management system (67.35%), and were unable to access the online course materials (71.43%) [5].

The study by Makaju S et al. reported that 84% of the 206 medical and dental students surveyed found virtual anatomy classes unhelpful for studying anatomy. The students encountered difficulties in studying dissection, cadaveric, and embryological structures through virtual classes [13].

Moreover, the lack of physical presence necessitates a higher level of motivation, discipline, and self-direction from students, as there is less external structure to guide them. The absence of social cues, such as body language and immediate feedback from peers and instructors, can also impede communication and reduce the richness of interpersonal interactions, potentially leading to a less engaging learning experience [14].

Traditional face-to-face lectures: strengths and weaknesses

Traditional face-to-face learning, despite the challenges posed during the pandemic, retains several key strengths that contribute to its continued preference among many students and educators. Traditional teaching methods provide the reality of being in a classroom, creating an environment that fosters learning.

The direct, in-person interaction inherent in this mode of learning fosters better student engagement. This setup makes it easier for students to ask questions, receive immediate feedback, and participate in discussions, leading to a more dynamic and interactive learning environment. Additionally, the physical presence of students and instructors can contribute to greater concentration span and more enthusiasm and interest in learning [15]. The structured nature of face-to-face learning is conducive to better understanding and memorization of subject material, particularly well-suited for practical sessions, where hands-on experience is crucial. Our study aligns with the findings of Sharma N et al. in Nepal, where 40.8% of students believed that regular classroom teaching cannot be substituted by online classes [11].

Study limitations

The study was conducted during the COVID-19 pandemic, a period characterized by unprecedented disruptions, and emotional and psychological stressors on both students and instructors. The unique circumstances of the pandemic may have influenced students' perceptions of online learning in ways that might not be as pronounced in a more stable, post-pandemic educational environment.

Differences in digital literacy play a critical role in determining a student's ability to effectively engage in online learning. With 88.67% of students possessing digital literacy, the remaining 11.33% may struggle to participate fully, skewing the results in favor of those more proficient with technology.

The type of device used may have introduced variability; while laptops or desktops are ideal, 95.6% of students relying on smartphones may encounter limitations in accessing and interacting with online course materials. Similarly, the quality of internet connections can vary widely, potentially disadvantaging students with less reliable or slower internet speeds.

The study did not extensively explore the instructors' adaptation to online teaching and pedagogical strategies employed by teachers, which could significantly influence student engagement and learning outcomes. Prior experience with online learning and individual learning styles may have affected the results.

The data collected in this study relied on self-reported responses from students. The study utilized a cross-sectional design, capturing data at a single point in time. The participants were enrolled using a convenience sampling technique, hence the findings may not be generalizable to all medical schools in India or globally. By acknowledging these limitations, future research can be better designed to address these gaps and provide more robust evidence on the efficacy of online synchronous teaching in medical education.

Challenges and recommendations

Institutions should provide access to reliable devices and high-quality internet connections for faculty and students, particularly for those who rely on smartphones or have limited internet access. Subsidies or loan programs could be explored to mitigate socioeconomic disparities.

Continuous professional development opportunities should be provided to instructors to enhance their ability to design and deliver engaging online courses. Training should focus on both technical skills and pedagogical strategies for online teaching.

To address the challenges of self-directed learning in an online environment, institutions should provide students with resources and guidance on time management, self-motivation, and effective study strategies. Additionally, incorporating structured milestones and regular check-ins can help maintain student discipline and progress.

To leverage the advantages of both synchronous online and traditional face-to-face learning, a hybrid model that combines elements of both should be considered. This approach can offer the flexibility and resource diversity of online learning while maintaining the engagement and interaction of face-to-face classes.

Continuous research should be conducted to evaluate the effectiveness of educational approaches in different contexts, particularly as technology and teaching methods evolve. Future studies should be multicentric, including a broader range of institutions and stakeholders to obtain a more comprehensive understanding of the effectiveness of different learning modalities.§

## Conclusions

Based on the findings of this study, it is concluded that while synchronous online learning provided a necessary alternative during the COVID-19 pandemic, it fell short of adequately supporting the study of complex subjects such as Physiology. Traditional face-to-face classes proved to be superior for these hands-on, detailed subjects, highlighting the limitations of online learning for certain aspects of medical education.

While virtual classes served as a temporary solution during the pandemic, they cannot fully replace the experiential learning that physical classes offer, particularly in disciplines requiring practical, tactile engagement. As such, a blended approach that combines the strengths of both modalities may be the most effective path forward.

## Appendices

Appendix 1 

**Table 5:** Study questionnaires on students' perceptions of traditional face-to-face lectures versus synchronous online classes.

Email	Age
Year of MBBS _I/ 2nd /3rd / 4th	Gender
You are invited to participate in a research study that aims to find medical students’ perceptions regarding traditional classes versus online classes, conducted by Dr. Smita Sorte. Department of Physiology, AIIMS Nagpur. You may contact Dr. Smita for any queries. Thank you for taking the time to consider this study.	
I give my full, free, voluntary consent to participate in the study. I confirm that I had the opportunity to ask questions. I understand that my participation is voluntary I permit PI to use my data. Yes / No	

Questions				
How do you access the internet?	cellular internet	Broadband	WI-FI	Dial-up Connection
Which device do you have?	Laptop	Desktop	Smartphone	I phone
Are you Digitally literate? - know learning management systems and basic computing programs such as email, Google apps, and publisher software (such as Word), as well as comfortable with helping other students troubleshoot basic technical difficulties	No	Maybe	Yes	
Do u consider yourself tech “savvy” or "techno-smart (Proficient in the use of technology, especially computers.?	No	Maybe	Yes	

Questions about traditional classes	1	2	3	4	5
The Instructor's presence affects your learning and motivates you to learn.	Strongly disagree	Disagree	Neutral	Agree	Strongly agree
The Learning Environment (Classroom and presence of colleagues) affects your active learning.	Strongly disagree	Disagree	Neutral	Agree	Strongly agree
Do you feel more disciplined and physically active in attending classroom lectures?	Strongly disagree	Disagree	Neutral	Agree	Strongly agree

Questions about synchronous online classes	1	2	3	4	5
The instructor not being physically present in a classroom benefits learners attending synchronous online sessions.	Strongly disagree	Disagree	Neutral	Agree	Strongly agree
Attending lectures alone in a room gives me more freedom and stimulates my learning.	Strongly disagree	Disagree	Neutral	Agree	Strongly agree
Do you feel you need to be more motivated, disciplined, and self-directed when attending synchronous online lectures?	Strongly disagree	Disagree	Neutral	Agree	Strongly agree
Do you feel more personal and individualized in the synchronous online course than face-to-face?	Strongly disagree	Disagree	Neutral	Agree	Strongly agree
Do you find it difficult to ask queries to the synchronous online instructor, as communication is often very impersonal?	Strongly disagree	Disagree	Neutral	Agree	Strongly agree

Question comparing two methods	Traditional classroom (Face to Face) Lectures	Synchronous online video lectures	Both
In which method is the concentration span greater?			
In which method do you get enough time to take notes?			
In which method is it more feasible to ask questions in real-time?			
Which method of engagement during the class is better?			
In which method can a variety of resources/ study materials be used?			
In which method can immediate feedback be given to the teachers?			
In which method class attendance can be easily given?			
Which method is more effective to make you feel more responsible regarding your exams?			
Which method is more convenient for studying at your own pace?			
Which method has more distracters?			
Which method generates more enthusiasm/interest in learning?			
Which method helps you to memorize relevant information			
Which method creates a better understanding of the subject?			
Which method do you prefer for theory lectures?			
Which method do you prefer for practical classes?			
Which method do you prefer for a tutorial?			
Which method do you prefer for case discussion?			
